# Attentional Performance, Age and Scholastic Achievement in Healthy Children

**DOI:** 10.1371/journal.pone.0032279

**Published:** 2012-03-29

**Authors:** Mireille Trautmann, Florian Daniel Zepf

**Affiliations:** 1 Department of Psychology, Albert Ludwigs University, Freiburg im Breisgau, Germany; 2 Department of Psychology, University of Leipzig, Leipzig, Germany; 3 Department of Child and Adolescent Psychiatry, Psychosomatics and Psychotherapy, University Hospital of the RWTH University, Aachen, Germany; 4 Institute for Neuroscience and Medicine, Jülich Research Centre, Jülich, Germany; 5 JARA Translational Brain Medicine, Aachen, Jülich, Germany; University of Leicester, United Kingdom

## Abstract

**Background:**

Attentional processes in children play a critical role in daily school demands and accomplishments. Studies on the association of attentional processes with school achievement and age in healthy school children are scarce. The aim of the present study was to identify correlations between dimensions of attentional performance, scholastic achievement and age.

**Methodology/Principal Findings:**

An extensive testing battery was used to assess a wide range of attentional dimensions. A principal component analysis revealed three factors that are related to attentional performance (distractibility, lapses of attention, cognitive speed). Age was negatively associated with distractibility, lapses of attention and cognitive speed, indicating that distractibility and lapses of attention decreased with age in healthy children and resulted in lower cognitive speed.

**Conclusions/Significance:**

Attentional processes in healthy children should be measured in relation to distractibility, lapses of attention and cognitive speed.

## Introduction

Attentional processes are the foundation for a variety of cognitive functions, such as perception, learning, the integration of information to establish working memory processes, and the active recall and integration of information [1], [2]. Attentional processes are important for daily intellectual performance, and they are essential for the acquisition of competencies, such as reading, spelling, and calculating [3], [4], [5], [6], [7]. In this context, it is necessary to differentiate between bottom-up and top-down attentional processing. Bottom-up processing is characterized by attending to an expected or unexpected external stimulus, whereas top-down processing reflects the control of internal processing, such as focusing on a specific task or internal process. Recalling information, for example, represents a top-down process. These processes allow to focus memory during the encoding and recall of information and integrate memory content into one schema [8]. For these reasons, attentional processes are indispensable for scholastic achievement.

Attentional processes are apparent in the context of the intensity and selectivity of stimuli, which can be thought of as quantitative and qualitative processing [9]. Van Zomeren and Brouwer [10] defined the qualitative aspect as the level of complexity and selectivity and the quantitative aspect, which is independent of task modality, as the intensity of attention on an explicit task. The authors found that vigilance and sustained attention require a lower level of mental effort when compared to active orientation and/or neglect over time. Vigilance and sustained attention can be used as measures of attentional intensity [10], [11]. However, independent of modality, both qualitative and quantitative attentional aspects are essential for adequately performing daily tasks.

Other concepts assume a supervisory attentional system that controls attentional processes that are responsible for focusing on an object [2], [9]. This approach is mostly used in the context of neurological diseases, such as disorders related to the frontal lobe of the brain. These models are mostly used in clinical contexts, but there are also general psychological models of attentional processes, such as the bottleneck-theory, that explain why specific information is focused on while other information is ignored [12], 13,14.

To focus on a specific task, one must neglect distractions that could trigger an external or internal attentional shift. For this reason, the active inhibition of distracters is required, and ignoring distractions while focusing on a specific task are separate subcomponents of attentional processes [15]. Above all, the inhibition of external distractions is essential to perceive and integrate new information that also requires working memory [2], [16], [17]. Földényi et al. [18], [19] used an adult testing battery to assess attentional performance in children. The testing battery for attentional performance (TAP) provides reliable, valid, and objective results in relation to subcomponents of attentional processes in adults and children [18], [19]. A specific TAP setup for the use in children (KITAP) was developed to assess attentional performance. The procedure was developed in accordance with the adult version and was based on the quantitative and qualitative features of Van Zomeren & Brouwer's neuropsychological model [10]. The KITAP is a standardized tool with exceptional psychometric properties and has been used in recent neuropsychological research on children with neuropsychiatric disorders such as ADHD [20], [21], [22] as well as in children who received a liver transplant [23] and children with motor coordination impairments [24]. In addition, as regards its clinical validity the KITAP was shown to be a good predictor for deficits as found attention deficit hyperactivity disorder (ADHD) in children aged 7 to 10 years, but was not sufficient as a single diagnostic tool [25]. A further study indicated that assessment of attentional functions using the KITAP helped to discriminate between children with and without ADHD (combined type), but the discriminative power was task-dependent and also depended on processing demands [21]. Moreover, the KITAP is a reliable application for cross-cultural assessment of attentional performance as indexed by findings in Syrian school-aged children [26].

Scholastic achievement is positively correlated with attention-related capabilities and the development of attentional processes. However, in most investigations, attentional performance has been assessed using teacher and parent ratings of children's ability to focus and shift attention. Attentional deficits in children have also been assessed with teacher and parent observations [27], [28], [29]. A major flaw in these studies is that the ratings of attentional performance were carried out by teachers and parents, thereby introducing a risk of rater bias. While there appears to be an association between attentional processes and scholastic performance, the specific aspects of attentional performance that are associated with scholastic achievement are unknown. An objective and reliable method of assessing these aspects is needed. Furthermore, age-related influences on attentional performance and scholastic achievement are unclear. The present study investigated this relationship by assessing cognitive speed (CS), distractibility (DS) and lapses of attention (LA) with the KITAP test. The sample included healthy children between 6.1 and 10.69 years old (see Methods for details). Scholastic achievement was assessed by school grades in different subjects (German and Mathematics). We expected to find correlations between age and aspects of attentional performance, and school grades, whereas we did not expect to find an association between scholastic achievement and age.

## Results

### Association between age, attentional performance and scholastic achievement

**Figure 1:**
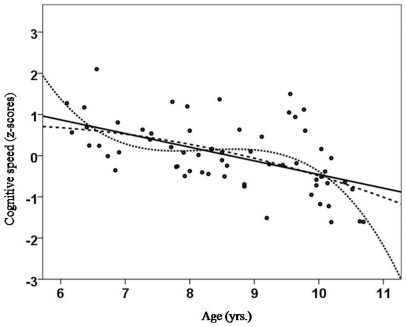
Correlation between age (yrs.) and cognitive speed as assessed by the KITAP (z-scores) [**18]**, [33****]. A linear trend indicated a reduction in cognitive speed with increasing age, a quadratic trend indicated a sudden decrease in cognitive speed at the age of 10 years, and a cubic trend was indicative of a stepwise decrease in cognitive speed (decrease – plateau – decrease) for the age range of 6 to 10 years.

**Figure 2:**
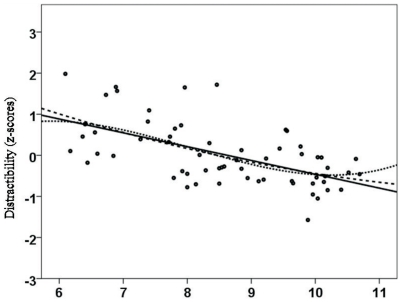
Correlation between age (yrs.) and distractibility as assessed by the KITAP [**18]**, [33****]. A linear trend was indicative of a reduction in distractibility with increasing age. There was no quadratic trend, and a cubic trend was indicative of a plateau (decrease – plateau) for ages greater than 10 years.

**Figure 3:**
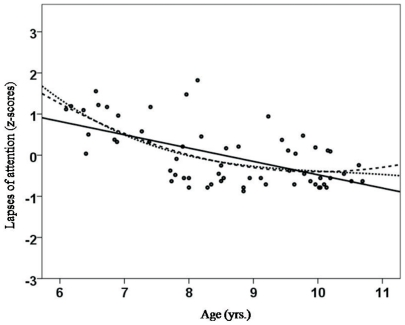
Correlation between age (yrs.) and lapses of attention as assessed by the KITAP [**18]**, [33****]. A linear trend was indicative of a reduction of lapses of attention with increasing age; a quadratic trend, but no cubic trend, was indicative of a plateau (decrease – plateau) starting at the age of 8 years.

**Figure 4:**
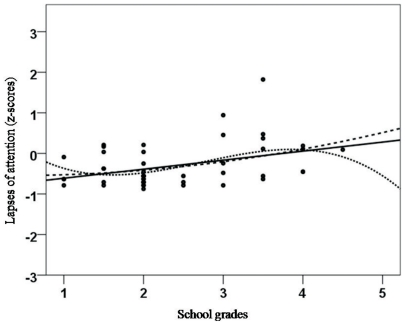
Correlation between scholastic achievement (school grades in German) and the z-scores of lapses of attention as assessed by the KITAP [**18]**, [33****]. A linear trend was indicative of an increase in lapses of attention with a decline in school grades (1 = “very good”; 6 = “worst”), and a quadratic and cubic trend was indicative of a plateau starting between the school grades 1 and 2.

As hypothesized, there were significant correlations between age and the three aspects of attentional performance (CS, DS and LA). CS and age were negatively correlated and followed a linear (r_xy_ = −0.532; *p*<0.01; see Fig. 1), quadratic (r_xy_ = −0.537; *p*<0.01) and cubic trend (r_xy_ = −0.541; *p*<0.01). As expected, we did not detect significant associations between age and scholastic achievement in our sample of healthy children. DS was negatively correlated with age (linear: r_xy_ = −0.584, *p*<0.01; quadratic: r_xy_ = −0.578, *p*<0.01; cubic: r_xy_ = −0.57, *p*<0.01; see Fig. 2). Lapses of attention (LA) were negatively correlated with age (linear: r_xy_ = −0.588, *p*<0.01; quadratic: r_xy_ = −0.57, *p*<0.01; cubic: r_xy_ = −0.551, *p*<0.01; see Fig. 3). Correlation analyses also indicated that all school grades correlated slightly with the three attention dimensions, but only German grades and lapses of attention correlated significantly (linear: r_xy_ = 0.388, *p*<0.01; quadratic: r_xy_ = 0.396; cubic: r_xy_ = 0.386; see Fig. 4). Male and female participants did not differ significantly in CS (F = 2.050; *p* = 0.157), DS (F = 0.629; *p* = 0.629) and LA (F = 0.000; *p* = 0.987).

## Discussion

The main results of this study are that the independent attentional dimensions, cognitive speed, distractibility, and lapses of attention, decreased with age. In other words, the data were indicative of improved attentional performance with increasing age and an associated development of resistance to distractibility, fewer lapses of attention, and a reduction in cognitive speed. Most studies assume that cognitive speed is highly correlated with attention in children. However, the present investigation indicates that specific aspects of attentional performance, which are indexed by distractibility and lapses of attention, correlate with age in healthy children.

A relevant factor in studies of attention-related features is the potential impact of motivational processes [30], which is particularly important among children. Motivational aspects, such as self-discipline, may also have an influence on scholastic achievement, which is highly correlated with self-control and attention [31]. For this reason, the secondary effects of self-control and self-monitoring must also be taken into consideration. Lapses of attention have been shown to be a reliable measure of attention-related processes in children with dyslexia [3]. Particularly among children, attention is related to age rather than intellectual abilities. Interventions that target attention problems at school should be evaluated as a potential avenue for improving scholastic performance [29].

Decreased lapses of attention and reduced distractibility with increasing age are closely related to the models of attentional processes that were outlined in the introduction [12], [13], [14]. The present study adds to the existing evidence of the development of attention-related features as children mature from childhood to adolescence. Four projection systems that relate to the modulation of intensity of attentional performance are of particular relevance. These systems appear to play a decisive role on the level of activation in attentional processes [32]. The relevant cortical and subcortical brain areas include the cholinergic regions of the basal forebrain, the noradrenergic regions of the locus coeruleus and dopaminergic forebrain bundle and the serotonergic raphe nucleus. With respect to functional connectivity to other brain areas, the anterior cingulated cortex (ACC), a brain region associated with both affective and cognitive processing, undergoes significant changes throughout development from childhood to adolescence to adulthood. A recent study examined the resting state functional connectivity of the ACC and found that children had diffuse functional connectivity, whereas adolescents revealed intermediate connectivity patterns [33]. Adults showed more distinct and focal patterns in their functional connectivity of the ACC [33]. With respect to brain development and the associated processes involving synaptic pruning, developmental aspects are likely to influence attentional performance during these maturational periods.

The present study has several limitations. Assessments of scholastic achievement by school grades in German and mathematics may not reflect all aspects of scholastic life in healthy children. Moreover, selection bias (i.e. that only students with better grades agreed to participate) must be taken into account although the spectrum of school grades in the present sample ranged from 1 to 5 (scale: 1 = “very good”, 6 = “worst”). Finally, although the KITAP provides a reliable way of measuring attentional performance, other methods of assessment may be taken into consideration in future studies.

In summary, the present study provides preliminary evidence that aspects of attentional performance improve with age, possibly at the cost of reduced cognitive speed.

## Materials and Methods

### Sample

Data were obtained from 61 healthy school children, between 6.1 and 10.69 years old (mean = 8.58 years; SD = 1.32), who attended a German primary school. Exclusion criteria were attentional and motor deficits as well as neurological and psychiatric conditions, which were assessed during parent interviews. Prior to participation in the study parents were asked if their children had a traumatic brain injury or intellectual disabilities, and such children were not included into the study. Intellectual capacity was not assessed in detail as the sample consisted of healthy children without any reported behavioral or school problems. In addition the parents were asked if their children had behavioral problems or other acute diseases. Children with severe untreated vision impairments (children with glasses or lenses) or hearing impairments did not participate in the study. The study was approved by the local school authorities (Oberschulamt, Freiburg im Breisgau, Germany) and carried out in accordance with the Helsinki Declaration. Parents, the head of the primary school and children gave their written informed consent to participate in the study. Characteristics of the study sample are provided in Table 1 and Table 2.

**Table 1:** Characteristics of the study sample (n = 61) as indexed by age (yrs., mean +/− standard deviation [SD]).

	Females	Males
*n*	33	28
*age*	8.56 (1.31)	8.61 (1.36)

**Table 2:** Sex and age distribution of the study sample (n = 61) as indexed by age groups (yrs., mean +/− standard deviation [SD], range).

**Age groups**	6 (n = 11)	7 (n = 10)	8 (n = 15)	9 (n = 13)	10 (n = 12)
**Mean ± SD**	6.54±0.27	7.68±0.24	8.46±0.30	9.59±0.28	10.25±0.24
**Range**	6.1–6.9	7.27–7.96	8.00–8.94	9.12–9.96	10.02–10.69
**Gender (M/F)**	5/6	5/5	7/8	5/8	7/5

### Assessment of attentional performance

Sub-dimensions of attentional performance were assessed using the KITAP [34]. The KITAP is easy to follow for children, and the testing battery has exceptional psychometric properties [18], [19], [34]. There was no special version used for children aged 6–7 and 8–10 years, which would have been of interest but which would have also resulted in the need of significantly larger samples. According to the developers the TAP has a split-half reliability of 0.55 to 0.97. For statistical analyses, median and standard deviation of reaction times were used. Omissions (OMs) and errors (ERs) were also included, and specific thresholds were defined to exclude outliers (see Tables 3 and 4).

**Table 3:** Parameters of attentional performance in the KITAP [19], [34] as indexed by the median (MD) of reaction time (RT), standard deviation (SD) of RT, error performance (ER) and omissions (OM).

MD of RT	SD of RT	ERRORS	OM
Alertness	Distractibility	Flexibility	Scanning
Distractibility	Visual Scanning		Go/No-go
Sustained attention	Go/No-go		Divided Attention
	Flexibility		

**Table 4:** Thresholds for exclusion of outliers for the median (MD) of reaction time (RT) in milliseconds (ms), with standard deviations (SD) for different subtests of the KITAP [19], [34].

Test	Threshold MD	Threshold SD
*Distractibility*	200 ms< = MD< = 800 ms	SD< = 400 ms
*Alertness*	200 ms< = MD< = 400 ms	SD< = 120 ms
*Sustained Attention*	400 ms< = MD< = 1000 ms	SD< = 300 ms
*Flexibility*	500 ms< = MD< = 2000 ms	SD< = 1000 ms
*Divided Attention*	600 ms< = MD< = 1100 ms	
*Go/No-go*	200 ms< = MD< = 800 ms	SD< = 200 ms
*Visual Scanning*	MD< = 19000 ms	SD< = 19000 ms

A confirmatory factor analysis was conducted to identify the relevant attentional domains assessed in the KITAP. The analysis showed that CS, DS and LA were the main dimensions of attention that explained most of the intraindividual variance. CS was accumulated by the median (MD) of the reaction times (RT) of the KITAP subtests *alertness, distractibility* and *sustained attention* with the following factor loads: MD of RT (alertness): 0.79; MD of RT (distractibility): 0.738; and MD of RT (sustained attention): 0.708. Factor loads for DS were also identified: SD of RT (Go/No-go): 0.738; SD of RT (visual scanning): 0.717; SD of RT (flexibility): 0.271; SD of RT (distractibility): 0.61; and errors (flexibility): 0.683. Factor loads for LA were 0.535 for omissions during divided attention, 0.57 for omissions in the Go/No-go task, and 0.511 for omissions during the visual scanning task.

### Assessment of school achievement

School grades in the subjects “German” and “Mathematics” were used to assess scholastic achievement (scale: 1 = “very good”, 6 = “worst”). These two subjects were chosen as they are taught throughout the entire school period in Germany, which would also allow future follow-up assessments. In addition, in the first school year students only get grades in the subjects “German” and “Mathematics”. Moreover, these particular subjects cover different aspects of scholastic performance, i.e. as regards verbal capabilities and mathematical reasoning.

To determine the reliability and internal consistency, Cronbach's alpha (0.915) and Guttman split-half reliability were computed (r (tt) = 0.87). A Kolmogorov-Smirnov goodness-of-fit test indicated that the data were normally distributed (t = 0.108; *p* = 0.086). The items were loaded on a single factor, which was derived by a confirmatory factor analysis, that explained 73.35% of the variance.

### Data analysis

Data analysis was performed using the SPSS software package (SPSS, Chicago). The level of statistic significance was set at *p*<0.05. Because of an explorative approach, significant *p*-values were not corrected in terms of an alpha adjustment. Associations between scholastic achievement, attention and age were examined with a dimensional approach using two-tailed bivariate Pearson's product-moment correlations. To make the results comparable, z-standardized values for the three attentional dimensions were used for correlation analyses. Other KITAP test parameters were analyzed using raw values. Differences in CS, DS and LA between sex were compared using Levene tests for variance homogeneity, indicating no significant differences.

## References

[1] Sturm W, Zimmermann P (2000). Aufmerksamkeitsstörungen.. Lehrbuch der klinischen Neuropsychologie.

[2] Oberauer K (2006). Is the focus of attention in working memory expanded through practice?. J Exp Psychol Learn Mem Cogn.

[3] Davis C, Coltheart M (2002). Paying attention to reading errors in acquired dyslexia.. Trends Cogn Sci.

[4] Facoetti A, Turatto M (2000). Asymmetrical visual fields distribution of attention in dyslexic children: a neuropsychological study.. Neurosci Lett.

[5] Facoetti A, Paganoni P, Lorusso ML (2000). The spatial distribution of visual attention in developmental dyslexia.. Exp Brain Res.

[6] Facoetti A, Paganoni P, Turatto M, Marzola V, Mascetti GG (2000). Visual-spatial attention in developmental dyslexia.. Cortex.

[7] Ashkenazi S, Rubinsten O, Henik A (2009). Attention, automaticity, and developmental dyscalculia.. Neuropsychology.

[8] Ciaramelli E, Grady C, Levine B, Ween J, Moscovitch M (2010). Top-down and bottom-up attention to memory are dissociated in posterior parietal cortex: neuroimagingand and neuropsychological evidence.. J Neurosci.

[9] Shallice T, Burgess P, Baddeley A, Weiskrantz L (1993). Supervisory control of action and thought selection.. Attention: Selection, Awareness and Control: A Tribute to Donald Broadbent.

[10] Van Zomeren AH, Brouwer WH (1994). Rehabilitation of attentional impairments. Clinical Neuropsychology of attention.

[11] Posner MI, Walker JA (1987). How do the parietal lobes direct covert attention?. Neuropsychologica.

[12] Treisman A, Fearnley S (1969). The Stroop test: selective attention to colours and words.. Nature.

[13] Treisman AM (1969). Strategies and models of selective attention.. Psychol Rev.

[14] Treisman AM, Riley JG (1969). Is selective attention selective perception or selective response? A further test.. J Exp Psychol.

[15] Andersen SK, Müller MM, Hillyard SA, Posner MI (2011). Tracking the allocation of attention in visual scenes with steady state evoked potentials.. Cognitive neuroscience of attention. 2nd ed.

[16] Oberauer K (2003). Selective attention to elements in working memory.. Exp Psychol.

[17] Geier CF, Garver K, Terwilliger R, Luna B (2009). Development of working memory maintenance.. J Neurophysiol.

[18] Földényi M, Tagwerker-Neuenschwander F, Giovanoli A, Schallberger U, Steinhausen H-C (1999). Die Aufmerksamkeitsleistungen von 6–10jährigen Kindern in der TAP.. Zeitschrift für Neuropsychologie.

[19] Földényi M, Giovanoli A, Tagwerker-Neuenschwander F, Schallberger U, Steinhausen H-C (2000). Reliabilität und Retestreliabilität der Testleistungen von 7-10jährigen Kindern in der computergestützten TAP.. Zeitschrift für Neuropsychologie.

[20] Hellwig-Brida S, Daseking M, Keller F, Petermann F, Goldbeck L (2011). Effects of methylphenidate on intelligence and attention components in boys with attention-deficit/hyperactivity disorder.. J Child Adolesc Psychopharmacol.

[21] Kaufmann L, Zieren N, Zotter S, Karall D, Scholl-Burgi S (2010). Predictive validity of attentional functions in differentiating children with and without ADHD: a componential analysis.. Dev Med Child Neurol.

[22] Nicolescu R, Petcu C, Cordeanu A, Fabritius K, Schlumpf M (2010). Environmental exposure to lead, but not other neurotoxic metals, relates to core elements of ADHD in Romanian children: performance and questionnaire data.. Environ Res.

[23] Kaller T, Langguth N, Ganschow R, Nashan B, Schulz KH (2011). Attention and executive functioning deficits in liver-transplanted children.. Transplantation.

[24] Michel E, Roethlisberger M, Neuenschwander R, Roebers CM (2011). Development of cognitive skills in children with motor coordination impairments at 12-month follow-up.. Child Neuropsychol.

[25] Drechsler R, Rizzo P, Steinhausen HC (2009). Zur klinischen Validität einer computergestützten Aufmerksamkeitstestbatterie für Kinder (KITAP) bei 7- bis 10-jährigen Kindern mit ADHS.. KIndheit und Entwicklung.

[26] Sobeh J, Spijkers W (2011). Development of attention functions in 5- to 11-year-old Arab children as measured by the German Test Battery of Attention Performance (KITAP): A pilot study from Syria.. Child Neuropsychol.

[27] Muris P (2006). Relation of attention control and school performance in normal children.. Percept Mot Skills.

[28] Breslau N, Breslau J, Peterson E, Miller E, Lucia VC (2009). Change in teachers' ratings of attention problems and subsequent change in academic achievement: a prospective analysis.. Psychol Med.

[29] Breslau J, Miller E, Breslau N, Bohnert K, Lucia V (2009). The impact of early behavior disturbances on academic achievement in high school.. Pediatrics.

[30] Chang F, Burns BM (2005). Attention in preschoolers: associations with effortful control and motivation.. Child Dev.

[31] Duckworth AL, Seligman ME (2005). Self-discipline outdoes IQ in predicting academic performance of adolescents.. Psychol Sci.

[32] Parasuraman R, Warm JS, See JE, Parasuraman R (1998). Brain systems of vigilance.. The attentive brain.

[33] Kelly AM, Di Martino A, Uddin LQ, Shehzad Z, Gee DG (2009). Development of anterior cingulate functional connectivity from late childhood to early adulthood.. Cereb Cortex.

[34] Zimmermann P, Gondan M, Fimm B (2004). Testbatterie zur Aufmerksamkeitsprüfung für Kinder (KITAP).

